# No Cognitive Scarring Over 20 Years in Recurrent Depression: Bidirectionality of Cognitive Functioning and Depression

**DOI:** 10.32872/cpe.20989

**Published:** 2026-08-31

**Authors:** Joost Gülpen, Amanda M. Legemaat, Eva A. M. van Dis, Gert J. Geurtsen, Ellie M. Wekking, Huibert Burger, Damiaan A. J. P. Denys, Claudi L. H. Bockting

**Affiliations:** 1 Department of Psychiatry, Amsterdam UMC, Amsterdam, The Netherlands; 2 Amsterdam Public Health Research Institute, Amsterdam, The Netherlands; 3 GGZ InGeest Specialized Mental Health Care, Amsterdam, The Netherlands; 4 Amsterdam Neuroscience Research Institute, Amsterdam, The Netherlands; 5 Department of Medical Psychology, Amsterdam UMC, Amsterdam, The Netherlands; 6 Parnassia Group, Den Haag, The Netherlands; 7 Department of Primary and Long-term Care Medicine, University Medical Center Groningen, Groningen, The Netherlands; Philipps-University of Marburg, Marburg, Germany

**Keywords:** cognitive functioning, major depressive disorder, relapse, scarring, cognitive impairments, bidirectionality

## Abstract

**Background:**

Cognitive deficits are prevalent in major depressive disorder (MDD), span multiple cognitive domains, and often persist during remission. However, long-term associations between cognitive functioning and depression remain unclear. This study explores bidirectional associations using 20-year longitudinal data, providing follow-up twice as long as previously reported.

**Method:**

Individuals in remission of recurrent MDD underwent neuropsychological assessments at baseline (*n* = 137, 21-65 years) and 20-year follow-up (*n* = 33), covering multiple domains. Psychiatric assessments occurred at eight time points. Cox proportional hazard models and linear mixed-effects models tested bidirectional associations between cognitive functioning and depression. Group-level cognitive change was tested using paired sample *t*-tests and Bayesian analyses, and individual-level change using reliable change indices (RCI’s).

**Results:**

No cognitive domains significantly predicted time-to-depressive-relapse, the number of depressive episodes, or total time depressed over 20 years. Similarly, the number of episodes, total time depressed, and changes in depression severity did not significantly predict cognitive changes. At follow-up, we observed declines in speed of information processing (*p* < .001, *d* = -0.73, RCI: 32.1% deteriorated) and speed of memory processing (*p* = .015, *d* = -0.49, RCI: 39.3% deteriorated). Learning and memory improved (*p* < .001, *d* = 1.19, RCI: 28.6% improved). Working memory and executive functioning remained stable. Findings were not explained by selective drop-out.

**Conclusions:**

We found only minor long-term cognitive change, with no clear worsening or evidence of scarring and state effects. Cognitive functioning remained relatively stable, and we observed no link to depression trajectories over 20 years, providing a more optimistic outlook for depressed patients.

## Highlights

- Cognitive functioning did not predict depression trajectories over 20-years follow-up, twice as long as prior work.
- Similarly, changes in cognitive functioning were not predicted by depression trajectories.
- No state- or scarring effects were found, and cognitive functioning remained relatively stable.

Major depressive disorder (MDD) is one of the most prevalent mental disorders (Ferrari et al., 2013), characterized by high rates of recurrence and treatment resistance (Hardeveld et al., 2010; McIntyre et al., 2023), making it a leading cause of disease burden. Traditionally, MDD treatment and research have primarily focused on affective symptoms, often overlooking cognitive functioning. However, cognitive deficits are not merely a clinical epiphenomenon of an affective disorder but constitute an important symptom and central feature. Between 24% and 79% of adults with MDD are affected by cognitive deficits, with the prevalence varying based on clinical state and definitions used (Douglas et al., 2018; Gualtieri, 2008; Liu et al., 2023; Stainton et al., 2023; Tran et al., 2021). Moreover, cognitive deficits contribute to the societal costs and disability associated with depression, as they are linked to a reduced quality of life and diminished functioning (Cambridge et al., 2018; Evans et al., 2013; Lam et al., 2014).

Interestingly, objective measures of cognitive performance poorly align with depressed patients’ subjective experiences (Serra-Blasco et al., 2019). This underscores the importance of focusing on objectively measured cognitive functioning when examining longitudinal associations with depression. Nonetheless, objectively measured cognitive deficits in MDD span multiple cognitive domains, including memory and learning, attention, psychomotor speed, processing speed, and executive functioning (Douglas & Porter, 2009; Hammar & Årdal, 2009; Roca et al., 2015; Rock et al., 2014; Snyder, 2013; Wagner et al., 2012). These deficits are evident across different stages of depression: during first acute episodes (Ahern & Semkovska, 2017; Lee et al., 2012; Varghese et al., 2022), recurrent episodes (Kriesche et al., 2023), and, remarkably, in remission (Kriesche et al., 2023; Semkovska et al., 2019). In remission, both objective and subjective cognitive deficits often persist as common residual symptoms (Conradi et al., 2011; Minor et al., 2005; Semkovska et al., 2019), indicating that clinical remission often does not equate cognitive remission (Bortolato et al., 2016).

Several hypotheses have been put forward to explain the nature of cognitive deficits in depression (Allott et al., 2016; Ang et al., 2020; Douglas & Porter, 2009; Hammar et al., 2022). First, it is hypothesized that deficits accumulate progressively with repeated depressive episodes and persist during remission (*scar hypothesis*: depression → cognitive functioning). Alternatively, cognitive deficits may fluctuate with the severity of depressive symptoms and improve when the depression resolves (*state hypothesis*: cognitive functioning ↔ depression). Lastly, they could stem from a pre-existing vulnerability that remains constant throughout the course of depression, regardless of clinical state (*trait hypothesis*: cognitive functioning → depression). Importantly, these three hypothesized mechanisms are not mutually exclusive, may co-occur in time, or vary across individuals, cognitive domains, and the phases of depression (Ahern et al., 2025; Allott et al., 2016; Ang et al., 2020; Semkovska et al., 2019). There is at least partial and mixed empirical support for these hypotheses, based on, for example, family studies (Allott et al., 2016), premorbid studies (Allott et al., 2016), cross-sectional comparisons between MDD patients and healthy controls (Ahern et al., 2025; Rock et al., 2014; Snyder, 2013), and studies comparing individuals with first-episode depression to those with recurrent depression (Guan et al., 2025; Talarowska et al., 2015). However, as many of these findings rely on between-group comparisons, longitudinal designs with repeated measures within the same individuals remain essential. Longitudinal approaches help minimize potential confounding that may arise when comparing different patient groups at different stages of illness.

Although cognitive deficits are a well-established symptom over the course of depression, reverse causality is also possible after initial MDD onset, with cognitive deficits contributing to the chronic nature of depression. For instance, cognitive deficits predict poorer treatment response (Groves et al., 2018) and an increased risk of relapse (Alexopoulos et al., 2000; Maeshima et al., 2016; Schmid & Hammar, 2013), although other studies found no such associations (Majer et al., 2004; Reppermund et al., 2009; Wekking et al., 2012). The heightened relapse risk may be explained by cognitive deficits facilitating cognitive biases, dysfunctional beliefs, and rumination (Ahern et al., 2019). Recent population-based longitudinal studies, primarily in older adults, have reported bidirectional associations between cognitive functioning and depressive symptoms (Fong et al., 2025; Ma et al., 2025; Yin et al., 2024). However, evidence from clinician-verified clinical samples using extensive neuropsychological testing and focusing on bidirectionality across the recurrent depressive course rather than late-life cognitive decline remains limited. Understanding the nature of cognitive deficits and their consequences is important, given their associated burden, and can additionally help identify targets for treatment and prevention.

This study aims to disentangle long-term bidirectional associations between cognitive functioning and depression over 20 years, increasing our understanding of their development over time following MDD onset. Longitudinal data from the DELTA-study (Legemaat et al., 2023), including recurrently depressed individuals in remission, provides a unique opportunity to address these questions. To our knowledge, this study provides the longest follow-up on cognitive functioning in depression, extending over twice as long as previously reported (Hammar & Årdal, 2012; Sarapas et al., 2012). In summary, we aim to: (a) explore to what extent cognitive functioning prospectively predicts depression trajectories over 20 years; (b) explore to what extent depression trajectories predict changes in cognitive functioning over 20 years; and (c) explore changes in cognitive functioning over 20 years. Based on previous research indicating bidirectionality, we hypothesize that cognitive functioning not only prospectively predicts the course of depression, but conversely, is also influenced by the course of depression.

## Method

### Design and Sample

This study is a follow-up of the DELTA-study (Legemaat et al., 2023; Wekking et al., 2012), a randomized controlled trial (RCT) examining effects of preventive cognitive therapy (PCT) in remitted MDD with follow-ups after 3 months and 1, 2, 3, 5.5, 10, and 20 years. Participants were recruited in 2000, and the follow-up ended in 2022. At entry, participants were (a) at least 18 years old and (b) in remission from MDD for at least 10 weeks and at most 2 years. They experienced (c) two or more previous MDD episodes in the last 5 years and (d) had 17-item Hamilton Depression Rating Scale (HDRS-17) scores less than 10 (Hamilton, 1960). Exclusion criteria were organic brain damage, substance abuse, current or previous psychotic disorders, current or previous (hypo)mania, predominant anxiety disorder, recent electroconvulsive therapy, and recent or current psychotherapy.

### Procedure

Participants were screened using the Structured Clinical Interview for DSM–IV (SCID-I) and the HDRS-17. Participants who met the inclusion criteria were randomized to PCT (8 weekly group sessions) or treatment as usual (TAU; comprising standard care) and were invited to attend blinded follow-up assessments. Neuropsychological assessments were conducted at baseline and at the 20-year follow-up. Psychiatric assessments were conducted at baseline and 3-month, 1-, 2-, 3-, 5.5-, 10-, and 20-year follow-up. Procedures were approved by the institutional review board. Participants gave informed consent prior to participation and at follow-ups.

### Outcomes

#### Neuropsychological Assessment

Neuropsychological assessments consisted of Dutch versions of internationally adopted tests (see Supplementary Table 1), conducted at baseline and 20-year follow-up only. The Stroop Color and Word Test (SCWT) was used to assess the speed of information processing (trials I and II) and executive functioning (trial III; Lezak et al., 2004). To assess the speed of memory processing, we used four subtasks of the Memory Comparison Task (MCT; Lezak et al., 2004). Additionally, both the backward and forward subtests of the Digit Span Test (Lezak et al., 2004) were used to measure working memory. To assess learning and memory, the Stories Recall subtest of the Rivermead Behavioral Memory Test (RBMT) and the Dutch California Verbal Learning Test (CVLT) were used. The Stories Recall subtest of the RBMT is a measure of immediate recall, absolute delayed recall, and relative delayed recall (Lezak et al., 2004; Wilson et al., 1991). The CVLT measures components of immediate recall and delayed recall (Mulder et al., 1996). Additionally, at 20-year follow-up, participants self-reported cognitive problems using the WHODAS 2.0 (Üstün, 2010).

Raw test scores were converted to standardized scores (*T*-scores: *M* = 50, *SD* = 10) based on Dutch normative data, controlling for sex, age, and educational level. To minimize the number of tests, we calculated composite *T*-scores for five cognitive domains, at both time points by averaging *T*-scores of (sub)tests: (a) speed of information processing (SCWT trails I and II); (b) speed of memory processing (MCT); (c) working memory (Digit Span forwards and backwards); (d) memory and learning (CVLT and RBMT subtests); and (e) executive functioning (SCWT trial III). Participants were classed as impaired (*T*-scores ≤ 36 considered as clinical range) or non-impaired on all five domains separately (Hermans et al., 2024). Performance validity was tested at 20-year follow-up, using the Test of Memory Malingering (TOMM; scores ≥ 45 considered valid) and Amsterdam Short Term Memory Test (ASTM; scores ≥ 85 considered valid; Schmand et al., 1999; Tombaugh, 1997). Total scores and classifications based on manual-recommended cut-off scores were used for the performance validity tests.

#### Depressive Relapse, Episodes and Severity

At baseline, 3-month, 1-, 2-, 3-, 5.5-, and 10-year follow-up, current and past depressive episodes were measured using the SCID-I and at 20 years using the Structured Clinical Interview for DSM-5 (SCID-5; First et al., 2016). Relapse was defined as meeting MDD criteria again 2 months after remission, while meeting criteria within 2 months was considered a continuation of the previous episode. These assessments also provided data on time-to-relapse, total number of episodes, and total time depressed (in days). The term *depression trajectories* is used as a collective term for these course indicators (e.g., time-to-relapse, number of episodes) and does not represent a separate outcome. Interrater agreement of assessments up to 10-year follow-up was high (*K* = .94). The 20-year assessments were conducted by one clinician.

We used the HDRS-17 (Hamilton, 1960) to assess depressive symptom severity as an outcome across follow-up psychiatric assessments. This semi-structured interview covers affective, behavioral, and somatic symptoms over the past week. Seventeen items are scored on 3-point or 5-point scales, with total scores ranging from 0 to 52. The HDRS-17 has good psychometric properties (Trajković et al., 2011) and showed acceptable average internal consistency across the DELTA-study (α = .73). The Beck Depression Inventory (BDI), a self-report measure of depressive symptom severity (Beck et al., 1961), was used to describe baseline clinical characteristics.

### Statistical Analyses

Analyses were conducted in R (Version 4.3.2), using a two-sided *p*-value of < .05 to determine statistical significance. Due to the small sample size and resulting low statistical power, we considered all our analyses exploratory. Consequently, no corrections for multiple testing were applied, and no power analysis was performed. Non-response analyses were conducted to compare participants who completed both neuropsychological assessments with those who dropped out, on clinical, cognitive, and demographic baseline values. Comparisons used χ^2^-tests for categorical variables and independent-sample *t*-tests for continuous variables. No imputation was conducted. Handling of missing data differed per analysis. Cross-sectional analyses were conducted using available cases. For the mixed-effects model, all available repeated measurements were included. To validate similarity between treatment groups (PCT vs. TAU), we assessed differences on the five cognitive domains at follow-up using analysis of covariance, adjusting for baseline cognition and, in a second analysis, adding the number of previous episodes (< 4 vs. ≥ 4) and the interaction with treatment, in line with previous work (Legemaat et al., 2023). If no significant or substantial effects were found, participants would be analyzed as one group in subsequent analyses. Spearman's rank-order correlations were calculated between self-reported cognitive problems and objective cognitive functioning (*T*-scores). Sensitivity analyses were conducted, excluding participants who failed performance validity tests. Across analyses, models were estimated without additional covariates given the exploratory nature of the study and modest sample size.

#### Cognitive Functioning as Predictor of Depression Trajectories (Aim 1)

Baseline cognitive functioning as a predictor of depression trajectories over 20 years was assessed for each cognitive domain (Aim 1). Effects of cognitive functioning on time-to-relapse were tested using separate Cox proportional hazards models to appropriately handle time-to-event data with censoring and to allow the estimation of relapse risk over time. Additionally, impaired versus non-impaired groups with each domain separately were compared using Kaplan-Meier survival curves and log-rank tests. A generalized linear mixed model with zero-inflation negative binomial distributions was used to predict the number of depressive episodes measured at seven follow-ups over 20 years, using the *glmmTMB* package, to account for excess zeros. Effect estimates were expressed as incidence rate ratios (*IRRs*). Linear mixed-effects models were used to predict total time depressed measured at seven follow-ups over 20 years, utilizing the *lme4* package. In general, linear mixed-effects models were used as they account for repeated continuous outcomes and can handle unbalanced, partially missing data. Baseline cognitive domain score and time (follow-up wave) were included as fixed effects. Random intercepts for participants were specified to account for within-subject dependency across repeated assessments.

#### Depression Trajectories as Predictors of Cognitive Changes (Aim 2)

We used linear mixed-effects models to explore whether long-term depression trajectories were predictive of cognitive change over time (Aim 2). For this, we predicted changes in cognitive domains separately, between baseline and follow-up, by the number of depressive episodes, total time depressed, and changes in HDRS-17 scores over 20 years (operationalized as the difference between baseline and 20-year follow-up scores). In each model, time (baseline vs. follow-up), the respective depression trajectory variable, and their interaction were included as fixed effects. A random intercept for participant was specified to account for within-subject dependency across repeated assessments. The interaction term tested whether long-term depression burden was associated with differential cognitive change between baseline and follow-up.

#### Changes in Cognitive Functioning (Aim 3)

Group-level changes, defined as mean within-subject changes, in cognitive functioning (Aim 3) were examined using paired sample *t*-tests for individual neuropsychological test scores and cognitive composite domain scores. With these analyses, we aimed to test whether on average cognitive functioning changed over time. Given the small sample size for these analyses, additional Bayesian analyses were conducted as this can quantify support in favor of the null hypothesis, and not only against it, using *BayesFactor*. To assess individual-level changes (Aim 3), reliable change indices (RCI) were calculated and plotted, using the *JTRCI* package. RCI values greater than 1.96 or less than -1.96 were seen as reliable improvements or deteriorations; an RCI between these values indicated no change. Individual profiles were examined to determine significant declines into clinically impaired ranges (*T*-scores ≤ 36). In a sensitivity analysis we excluded participants failing performance validity tests (TOMM and/or ASTM).

## Results

### Sample Characteristics

**Figure 1:**
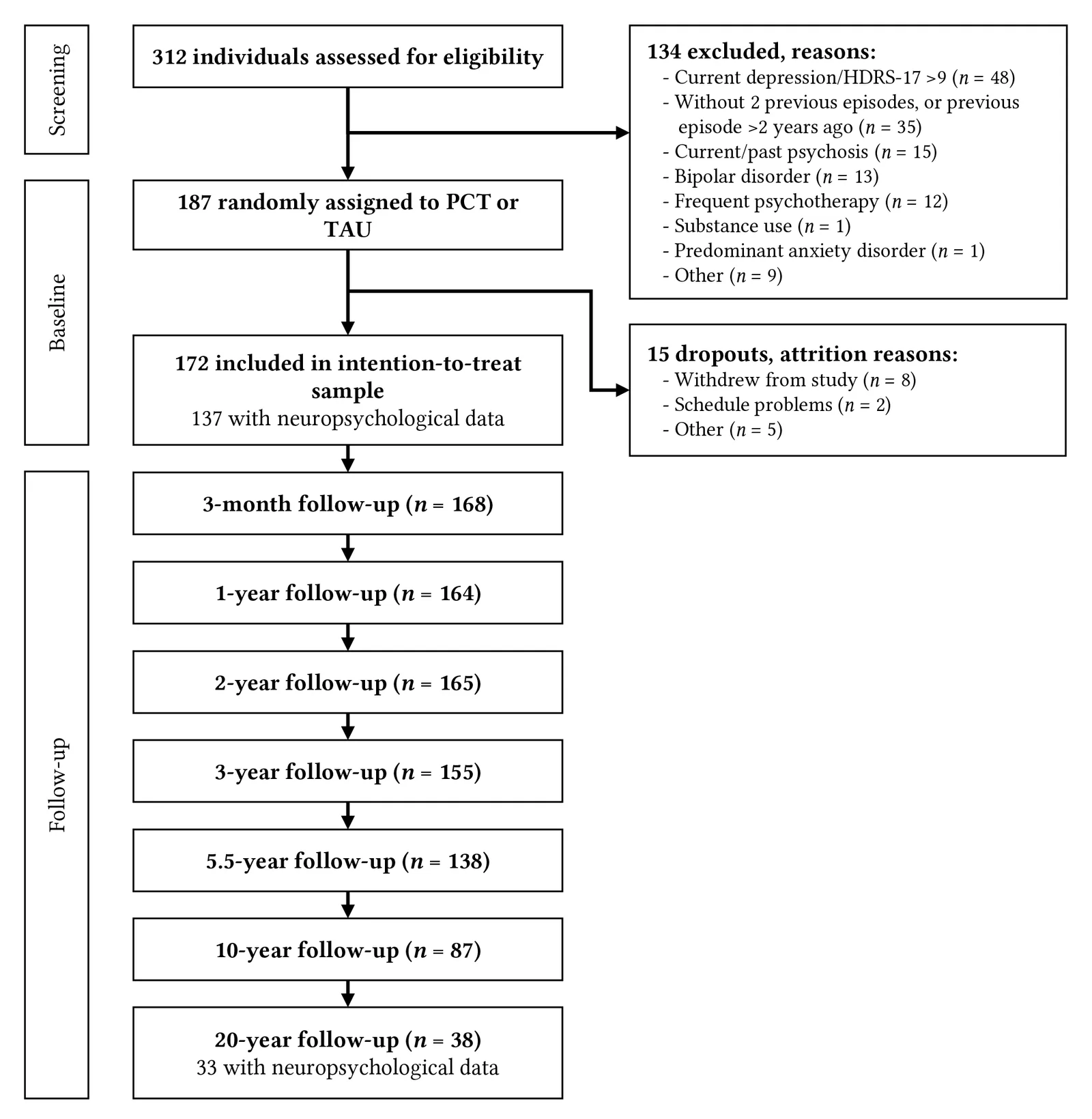
Flow Diagram of Participants From Baseline to 20-Year Follow-Up *Note.* HDRS-17 = 17-item Hamilton Depression Rating Scale; PCT = preventive cognitive therapy; TAU = treatment-as-usual.

Overall, 312 individuals were assessed for eligibility, and 172 were included, of whom 137 individuals aged 21-65 years (*Mdn* = 46) completed the baseline neuropsychological assessment (Figure 1). At 20-year follow-up, 38 individuals were evaluated, with 33 (19.2%) undergoing the neuropsychological assessment (PCT: *n* = 18; TAU: *n* = 15). In total, 28 individuals (16.3%) completed neuropsychological assessments at both baseline and 20-year follow-up. In a drop-out analysis, all clinical, cognitive, and demographic baseline values were highly similar between those assessed at both time points and those only seen at baseline (Supplementary Table 2), except for BDI scores (*p* = .022). Baseline characteristics for participants with cognitive data at baseline (*n* = 137) and follow-up (*n* = 33) are presented in Table 1. Individuals participating in the 20-year follow-up had a baseline average age of 44.8 years (*SD* = 7.55), with 78.8% female, 27.3% highly educated, and four previous depressive episodes (*IQR* = 3–6). The mean interval between neuropsychological assessments was 256 months (range = 251-259 months). At 20-year follow-up, no participants reported acquired brain injuries or neurodegenerative diseases. Over follow-up, one individual received electroconvulsive therapy; none underwent deep-brain stimulation. Performance validity tests at 20-year follow-up found no failures on the TOMM and seven failures (21.2%) on the ASTM. Treatment allocation had no significant effects on any cognitive domains, regardless of adding the number of previous episodes (< 4 vs. ≥ 4), and the interaction with treatment was not substantial (all *p*-values > .05; Supplementary Table 3). On average, participants with cognitive data at both time points (*n* = 28) had 3.29 (*SD* = 2.68) depressive episodes and were depressed for 93.81 weeks (*SD* = 157.77) over 20 years.

**Table 1:** Baseline Demographic and Clinical Characteristic of Included Participants

Variable	Baseline sample (*n* = 137)	20-year follow-up sample (*n* = 33)
Demographic characteristics		
Age, years (*M, SD*)	44.91 (9.38)	44.79 (7.55)
Gender, Female (*n*, %)	102/137 (74.45)	26/33 (78.79)
Married/cohabiting (*n*, %)	85/137 (62.04)	17/33 (51.52)
Education level (*n*, %)		
High	54/137 (39.42)	9/33 (27.27)
Middle	41/137 (29.93)	12/33 (36.36)
Low	42/137 (30.66)	12/33 (36.36)
Education level, years (*M*, *SD*)	14.02 (2.01)	13.90 (2.05)
Clinical characteristics		
Age of MDD onset, years (*M*, *SD*)	28.18 (12.69)	29.06 (12.72)
Months in remission (*M*, *SD*)	8.90 (6.44)	9.45 (7.22)
Number of previous episodes (*Mdn*, *IQR*)	4 (3-6)	4 (3-6)
>3 previous episodes (*n*, %)	114/137 (83.21)	26/33 (78.79)
Duration previous episode, months (*M*, *SD*)	7.20 (8.73)	7.46 (6.45)
Depressive symptoms, HDRS-17 (*M*, *SD*)	3.76 (3.03)	3.94 (3.23)
Depressive symptoms, BDI (*M*, *SD*)	11.92 (7.49)	9.96 (6.88)
Antidepressant usage (*n*, %)	72/137 (52.55)	12/33 (36.36)
Benzodiazepine usage (*n*, %)	17/137 (12.41)	7/33 (21.21)
Clinical cognitive impairment on ≥1 domain ^a^ (*n*, %)	77/137 (56.20)	19/33 (57.58)
Clinical cognitive impairments ^a^ (*n*, %)		
Speed of information processing	22/137 (16.06)	9/33 (27.27)
Speed of memory processing	35/137 (25.55)	14/33 (42.42)
Working memory	6/137 (4.38)	0/33 (0)
Memory and learning	39/137 (28.47)	2/33 (6.06)
Executive functioning	16/137 (11.68)	4/33 (12.12)

*Note.* Data are mean (*SD*), median (*IQR*), or *n*/*N* (%). BDI = Beck Depression Inventory; HDRS-17 = 17-item Hamilton Depression Rating Scale; *IQR* = interquartile range; MDD = major depressive disorder; *SD* = standard deviation.

^a^*T*-scores ≤ 36 are considered as cognitive impairments within clinical ranges.

### Cognitive Functioning as Predictor of Depression Trajectories

No significant effects of time-to-relapse (*n* = 137) were found for speed of information processing (*HR* = 0.99, 95% CI [0.97, 1.01], *p* = .343), speed of memory processing (*HR* = 1.01, 95% CI [0.99, 1.02], *p* = .546), working memory (*HR* = 0.99, 95% CI [0.96, 1.01], *p* = .271), learning and memory (*HR* = 1.00, 95% CI [0.98, 1.01], *p* = .637), and executive functioning (*HR* = 1.01, 95% CI [0.99, 1.03], *p* = .511). We only observed survival differences in the speed of information processing when comparing clinically impaired versus non-impaired groups (χ^2^(1, *N* = 137) = 6.1, *p* = .013), with a significantly higher probability of relapse amongst impaired participants (Supplementary Figure 1, for Kaplan-Meier curves per domain).

Speed of information processing (*IRR* = 0.99, 95% CI [0.98, 1.01], *p* = .369), speed of memory processing (*IRR* = 1.00, 95% CI [0.99, 1.02], *p* = .482), working memory (*IRR* = 0.99, 95% CI [0.97, 1.01], *p* = .336), learning and memory (*IRR* = 0.99, 95% CI [0.98, 1.01], *p* = .299) and executive functioning (*IRR* = 1.01, 95% CI [1.00, 1.02], *p* = .121) did not significantly predict the number of depressive episodes over 20 years (*n* = 137, observations = 866). Similarly, none of the cognitive domains significantly predicted the total time depressed over 20 years (*n* = 137, observations = 866): speed of information processing (β = -0.07, 95% CI [-0.14, 0.00], *p* = .065, partial *R*^2^ = 0.01), speed of memory processing (β = 0.00, 95% CI [-0.07, 0.07], *p* = .985, partial *R*^2^ = 0.00), working memory (β = -0.03, 95% CI [-0.10, 0.04], *p* = .438, partial *R*^2^ = 0.00), learning and memory (β = 0.04, 95% CI [-0.03, 0.11], *p* = .239, partial *R*^2^ = 0.00), and executive functioning (β = 0.04, 95% CI [-0.03, 0.11], *p* = .247, partial *R*^2^ = 0.00).

### Depression Trajectories as Predictors of Cognitive Changes

We explored whether the course of depression predicts changes in cognitive functioning over 20 years using linear mixed-effects models. However, the number of depressive episodes, total time depressed, and changes in HDRS-17 scores did not significantly predict changes in cognitive functioning over 20 years (all *p*s > .05; Supplementary Table 4). Sensitivity analyses excluding participants failing performance validity based on the ASTM did not change the results (all *p*s > .05; results not shown).

### Changes in Cognitive Functioning

Standardized *T*-scores and raw scores on neuropsychological tests and domains across time points are presented in Table 2. The majority of patients had *T*-scores in clinical ranges on one or more cognitive domains (56% at baseline; 58% at follow-up; see Table 1). There were significant declines in *T*-scores for speed of information processing (mean difference = -5.04; 95% CI [-7.73, -2.36], *p* < .001, *d* = -0.73) and speed of memory processing (mean difference = -5.08; 95% CI [-9.09, -1.08], *p* = .015, *d* = -0.49). A significant improvement was found for learning and memory (mean difference = 10.88; 95% CI [7.32, 14.44], *p* < .001, *d* = 1.19). No changes were found for working memory (mean difference = -0.02; 95% CI [-2.84, 2.87], *p* = .990, *d* = 0.00) and executive functioning (mean difference = -0.28; 95% CI [-3.41, 2.85], *p* = .855, *d* = 0.04). After conducting sensitivity analyses excluding seven participants failing performance validity tests, changes in speed of memory processing were nonsignificant (*p* = .097). Other effects remained unchanged (results not shown). Bayesian paired samples *t*-tests showed very strong evidence for changes in speed of information processing and learning and memory, and moderate evidence for speed of memory processing (Supplementary Table 5). For working memory and executive functioning, moderate evidence supported the null hypothesis (i.e., no change over time).

**Table 2:** Mean (SD) of Standardized and Raw Scores on Neuropsychological Tests at Baseline and 20-Year Follow-up Across Domains for Participants With Complete Neuropsychological Data at Both Follow-ups (n = 28).

Neuropsychological test and domain	Test score					Reliable Change Index		
	Baseline		20-year follow-up		*p* ^a^	Reliably deteriorated *n* (%)	No change *n* (%)	Reliably improved *n* (%)
	*T*-scores *M* (*SD*)	Raw scores *M* (*SD*)	*T*-scores *M* (*SD*)	Raw scores *M* (*SD*)				
Speed of Information Processing	45.65 (6.81)		40.61 (7.57)		< .001*	9 (32.14)	18 (64.29)	1 (3.57)
Stroop Color and Word Test – Trial I	45.51 (8.20)	44.75 (8.31)	39.36 (8.39)	51.21 (8.61)	< .001*	8 (28.57)	20 (71.43)	0 (0)
Stroop Color and Word Test – Trial II	45.79 (7.83)	58.07 (8.36)	41.86 (9.30)	65.25 (12.26)	.019*	8 (28.57)	18 (64.29)	2 (7.14)
Speed of Memory Processing	42.78 (11.22)		37.69 (13.51)		.015*	11 (39.29)	13 (46.43)	4 (14.29)
Memory Comparison Task – 1 Letter	38.47 (12.91)	27.96 (5.47)	38.01 (13.01)	42.93 (10.38)	.837	4 (14.29)	19 (67.86)	5 (17.86)
Memory Comparison Task – 2 Letters	43.43 (12.75)	39.43 (9.17)	45.48 (12.93)	52.25 (14.72)	.378	2 (7.14)	19 (67.86)	7 (25.00)
Memory Comparison Task – 3 Letters	44.34 (10.05)	50.14 (9.97)	33.74 (18.06)	75.82 (22.90)	< .001*	15 (53.57)	12 (42.86)	1 (3.57)
Memory Comparison Task – 4 Letters	44.86 (14.61)	68.82 (24.21)	33.54 (17.73)	92.21 (26.35)	.004*	10 (35.71)	17 (60.71)	1 (3.57)
Working Memory	49.25 (7.00)		49.23 (7.61)		.990	2 (7.14)	24 (85.71)	2 (7.14)
Digit Span Test – Digits forward	48.21 (8.62)	5.39 (0.92)	47.54 (9.81)	5.43 (1.07)	.751	3 (10.71)	23 (82.14)	2 (7.14)
Digit Span Test – Digits backward	50.29 (7.30)	4.46 (1.07)	50.93 (8.66)	4.71 (1.21)	.679	1 (3.57)	26 (92.86)	1 (3.57)
Learning and Memory	45.14 (10.34)		56.01 (12.96)		< .001*	0 (0)	20 (71.43)	8 (28.57)
Dutch California Verbal Learning Test								
Immediate recall	49.29 (28.14)	54.86 (11.56)	63.21 (25.68)	55.07 (11.02)	.001*	0 (0)	28 (100)	0 (0)
Delayed recall	48.93 (22.50)	12.43 (2.94)	60.71 (28.27)	13.50 (2.17)	.034*	3 (10.71)	18 (64.29)	7 (25.00)
Rivermead Behavioral Memory Test								
Immediate recall – subtest Stories	41.73 (11.00)	17.39 (5.75)	49.82 (11.72)	18.86 (7.10)	.003*	0 (0)	21 (75.00)	7 (25.00)
Absolute delayed recall – subtest Stories	40.54 (10.86)	14.41 (5.64)	52.50 (12.50)	16.25 (7.07)	< .001*	0 (0)	13 (46.43)	15 (53.57)
Relative delayed recall – subtest Stories	45.19 (13.78)	^b^	53.82 (13.57)	^b^	.020*	1 (3.57)	21 (75.00)	6 (21.43)
Executive Functioning	47.71 (10.21)		47.43 (9.65)		.855	3 (10.71)	22 (78.57)	3 (10.71)
Stroop Color and Word Test – Trial III	47.71 (10.21)	94.32 (22.43)	47.43 (9.65)	104.93 (25.24)	.855	3 (10.71)	22 (78.57)	3 (10.71)

*Note.* *M* = mean; *SD* = standard deviation.

^a^Results from paired-sample *t*-tests, comparing baseline and 20-year follow-up *T*-scores. ^b^No raw scores available as this *T*-score is calculated using the immediate and absolute delayed recall scores. *p ≤ .05.

**Figure 2:**
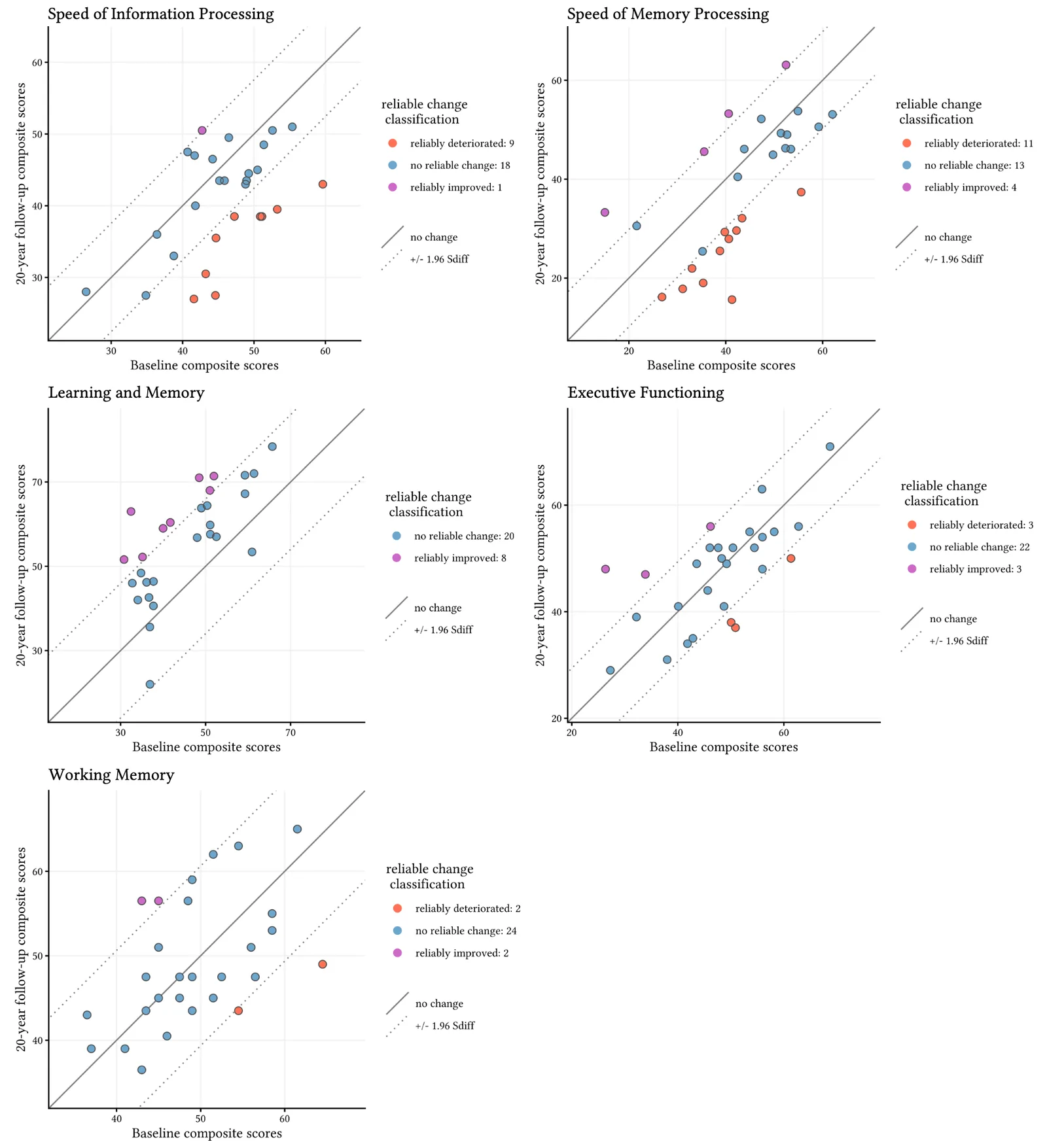
Reliable Change Index Plots of Composite T-Scores for the Five Cognitive Domains, Visualizing Individual-Level Change Between Baseline and 20-Year Follow-Up

Moreover, using RCIs, we observed no specific pattern of change in cognitive functioning across specific tests and domains (Table 2 and Figure 2), although most declines were seen for speed of information processing (32.1% reliably deteriorated) and speed of memory processing (39.3% reliably deteriorated).

Regarding the working memory, a majority of 85.7% remained unchanged. In contrast, no individuals deteriorated in learning and memory, and 28.6% significantly improved. On executive functioning, 78.6% showed no reliable change. Individual *T*-scores across cognitive profiles showed that most individuals who experienced declines in speed of information processing over 20 years also showed declines in speed of memory processing, and vice versa (Supplementary Table 6). In total, four of the 28 individuals showed a significant decline and fell into clinical ranges on speed of information processing, and 10 of the 28 individuals in speed of memory processing, while no such reliable changes were seen for other domains (Supplementary Table 6). Excluding participants failing performance validity tests did not considerably alter patterns of reliable change (Supplementary Table 7). Only five people did not relapse over 20 years, however, we did not observe different patterns among these individuals (Supplementary Table 8). At follow-up, 85.7% of individuals (24/28) self-reported cognitive problems: 42.9% minor (12/28), 28.6% moderate (8/28), and 14.3% severe (4/28). Only 2 of the 4 individuals reporting severe problems had *T*-scores in clinical ranges. Self-reported cognitive problems did not correlate with objectively measured cognitive domains at 20-year follow-up (all *p*-values > .05; Supplementary Figure 2). Boxplots showing baseline and 20-year follow-up cognitive functioning by domain are shown in Supplementary Figure 3.

## Discussion

In this longitudinal study, we explored cognitive change and bidirectional associations between cognitive functioning and depression over 20 years – a follow-up twice as long as currently reported – amongst individuals in remission from recurrent MDD. Importantly, in this at-risk group, cognitive functioning was not associated with prospectively assessed time-to-relapse nor with the number and duration of depressive episodes over 20 years. Likewise, while we could not completely rule this out due to the small sample, we found no indication of either scarring- or state-effects over 20 years. Our results, while explorative, challenge the assumption that cognitive deterioration due to scarring is an inevitable consequence of recurrent depressive episodes. Additionally, we found only limited cognitive change over 20 years and more importantly no marked deterioration. Therefore, our findings suggest that cognitive functioning in depression may remain relatively stable over extended periods, irrespective of the number of recurrences. Taken together, our observations may suggest that long-term cognitive changes and their bidirectional associations with depression may not be as pronounced as previously hypothesized, providing a more optimistic long-term outlook for depressed patients.

Furthermore, cognitive functioning did not predict the depression course over 20 years. None of the five domains, as measured using demographically corrected *T*-scores, were associated with time-to-relapse or the number and duration of episodes over 20 years. However, participants showing clinical impairments in speed of information processing were more likely to relapse compared to those without such impairments. This finding is noteworthy, as processing speed is one of the domains most affected in remitted MDD (Semkovska et al., 2019) and especially pronounced in those with recurrent episodes (Varghese et al., 2022). Cognitive functioning as a risk factor for relapse has been examined in only a limited number of studies, with mixed results. While some studies have found no significant associations (Majer et al., 2004; Reppermund et al., 2009; Wekking et al., 2012), others, in contrast to our findings, found support for memory impairments (Maeshima et al., 2016), and executive functioning impairments as predictors of relapse (Alexopoulos et al., 2000; Schmid & Hammar, 2013). However, previous studies differ considerably in terms of their samples (i.e. first episode vs. recurrent MDD, unipolar vs. bipolar, geriatric sample), follow-up periods, and cognitive domains assessed. Our study adds to this growing body of research by including a comprehensive neuropsychological and psychiatric assessment, with the longest follow-up and largest sample currently available. Taken together, cognitive functioning was not found to be a clinical marker of chronicity. Cognitive impairment itself, however, was found to persist over 20 years in this sample.

Surprisingly, we did not find any evidence for scarring- or state-like effects, as cognitive change was unrelated to changes in depression severity or the number and duration of depressive episodes over 20 years. In contrast, several meta-analytic studies in acute and remitted MDD reported worsening cognitive functioning or moderation of cognitive change by depressive symptom severity and the number of episodes (Ahern et al., 2025; Ahern & Semkovska, 2017; Semkovska et al., 2019). Nevertheless, our findings do in part align with current perspectives suggesting that such scar- and state effects are rarely universal but tend to be domain-specific and influenced by methodological factors, e.g. cognitive tests used, indices of depression assessed (e.g., symptom status vs. number of episodes), clinical status (remitted, first-episode vs. recurrently depressed) and study design. Population-based studies on this topic are very limited, with mixed results, and are mostly cross-sectional, reliant on brief cognitive assessments, or primarily focused on symptom levels and not clinician-verified diagnoses (de Nooij et al., 2020; Formánek et al., 2020; Zhu et al., 2022). However, a study using UK Biobank data, with validated diagnostic interviews and comprehensive cognitive assessments, similarly found no evidence that previous episodes or symptoms were associated with cognitive impairments in individuals with a previous MDD diagnosis (de Nooij et al., 2020). There are several possible explanations that could explain our observations. First, our remitted, recurrent sample reported a median of four previous episodes at baseline. Therefore, some potential scarring effects might have already occurred before baseline neuropsychological assessment. Indeed, there is evidence that cognitive impairment takes places early in the disease-course (Varghese et al., 2022). Second, our analyses suffered from limited statistical power as neuropsychological data at both time-points was available for only 28 participants.

Overall, while the majority of patients had *T*-scores in clinical ranges on one or more cognitive domains, we found no clear group-level worsening or improvement of cognitive functioning. Whereas both speed of information processing and speed of memory processing decreased over 20 years, changes are not clinically meaningful. Although not fully explained, we did observe a large improvement in learning and memory. Some small-to-moderate pro-cognitive change has been observed in this domain (Ahern et al., 2025), but it remains unclear why this finding differentiates from other domains. However, learning and memory had the highest number of participants with clinical impairments (28.5%) and had – apart from speed of memory processing – the lowest average *T*-score. This improvement may thus, at least in part, be attributed to a regression-to-the-mean effect. Interestingly, recurrently depressed individuals perform significantly worse on learning and memory tests and show the largest impairments on this domain, compared to first-episode individuals, indicative of a progressive decline (Varghese et al., 2022). Working memory and executive functioning were not subjected to group-level change and showed limited individual-level change using RCIs. RCIs did show that a subset of participants experienced reliable deteriorations, particularly in speed of information processing and speed of memory processing, underscoring inter-individual variability and, at least in part, contradicting group-level findings. It is possible that our observations are influenced by confounding factors associated with increased age given the long follow-up which may not have been fully captured, such as long-term treatment, medication or substance usage, hospitalization, or chronic somatic conditions. However, *T*-scores were used based on normative data across several age categories to correct for age-related cognitive decline, and we could not attribute cognitive changes to medication usage, time in remission or chronic somatic conditions. Nevertheless, our findings are somewhat reassuring as patients may worry about progressively worsening and long-lasting cognitive deficits following depression.

This study has several strengths. Composite scores from an elaborate neuropsychological test-battery were used to increase reliability compared to single tests, with corrections for age, sex and education level. Our design also allowed us to assess cognitive stability over 20 years in relation to depression – the longest follow-up currently available – and to examine longitudinal associations over eight measurements. Additionally, results could not be explained by differential drop-out. Lastly, we did not only examine cognitive change on a group-level, but also on an individual-level using RCIs.

Nevertheless, several limitations should be noted. First, we included individuals in remission from recurrent MDD, with at least 2 previous episodes, limiting generalizability. Relatedly, assessments of cognitive changes earlier in the illness course are more interpretable with regards to scar, trait and state hypotheses (Allott et al., 2016). Second, we lacked a healthy control group, which is necessary to adequately control for practice effects and relate changes in performance to clinical variables. We were thus unable to compare developmental trajectories of this sample with prior depressive episodes to change over time in a non-MDD sample. Third, given the exploratory character of our study due to insufficient sample size in our analyses, no corrections for multiple testing were conducted. As such, our results have limited precision and should be considered exploratory, requiring cautious interpretation. Fourth, cognitive functioning was assessed at only two time points over 20 years, limiting our ability to properly examine bidirectional associations between depression and cognition. As the interplay between these variables is likely dynamic, future studies should include more frequent repeated neuropsychological and psychiatric assessments over this timeframe, using different longitudinal approaches (e.g., cross-lagged or latent growth models) to better capture bidirectional effects. Fifth, this longitudinal follow-up study was part of an RCT, and some participants were actively seeking treatment or took part in a systematic intervention, although we found no effects of treatment allocation on cognitive functioning at 20 years. Lastly, we were unable to account for premorbid cognitive functioning or IQ in the current study, using an idiographic or intraindividual approach (Lezak et al., 2004). While we used a normative approach to compare individuals’ performance compared to demographically matched peers, this may not capture relative deficits compared to premorbid levels. Thus, some baseline impairments may be the result of low premorbid functioning. Conversely some impairments may be missed in individuals with higher premorbid cognitive functioning, while they may have experienced deteriorations from their premorbid levels.

In conclusion, in this exploratory study we found no clear evidence supporting cognitive functioning as a predictor of depression chronicity and recurrence over 20 years, apart from clinical impairments in the speed of information processing. Additionally, within this recurrent, remitted sample, we did not observe consistent scarring- or state-like effects on cognitive functioning. Participants in remission of recurrent MDD did not experience substantial cognitive decline over 20 years. However, the majority of patients showed persistent cognitive impairment over this time period. Although our findings are exploratory, and consequently the precision of our estimates is limited, they still suggest that the long-term cognitive impact of depression and their bidirectional associations may be less pronounced than previously thought. Large-scale longitudinal studies including individuals with clinician-verified MDD and extended follow-ups are warranted. Therefore, our results should be interpreted with caution. Nonetheless, given the considerable follow-up of our study, our findings offer a valuable perspective into cognitive functioning in depression and may provide some reassurance to patients suffering from recurrent MDD.

## Supplementary Materials

The Supplementary Materials contain the following items:

- **The preregistration for the study** (Gülpen et al., 2021S)
- **Supplementary tables and figures** (Gülpen et al., 2026S):
  - *Supplementary Table 1.* Concise description of the neuropsychological tests and the tasks administered.
  - *Supplementary Table 2.* Results from non-response analysis examining selective drop-out based on demographic, cognitive and clinical characteristics.
  - *Supplementary Table 3.* Results of ANCOVAs assessing differences between PCT and TAU on five cognitive domains at 20 year follow-up.
  - *Supplementary Table 4.* Outcomes from linear mixed-effects model of depression variables as predictors of cognitive functioning change over 20 year follow-up.
  - *Supplementary Table 5.* Results of Bayesian analyses of differences in five cognitive composite domains between baseline and 20 year follow-up (*n* = 28).
  - *Supplementary Table 6.* Individual reliable change indices and T-scores, of all participants with complete neuropsychological data at baseline and 20 year follow-up across five composite domains.
  - *Supplementary Table 7.* Results of reliable change index analyses after sensitivity analyses excluding participants failing performance validity.
  - *Supplementary Table 8.* Results of reliable change index analyses for participants not experiencing a relapse over 20 year follow-up.
  - *Supplementary Figure 1.* Kaplan-Meier survival curves of time-to-relapse comparing participants with clinical impairments on cognitive domains to those non-impaired.
  - *Supplementary Figure 2.* Correlation matrix showing associations at 20-year follow-up between self-reported cognitive problems and objective cognitive functioning.
  - *Supplementary Figure 3.* Boxplots showing baseline and 20-year follow-up cognitive functioning by domain.

GülpenJ.
LegemaatA. M.
van DisE. A. M.
GeurtsenG. J.
WekkingE. M.
BurgerH.
DenysD. A. J. P.
BocktingC. L. H.
 (2021S). Relapse, cognitive, and daily functioning in recurrently depressed individuals: DELTA study 20-year follow-up
[Preregistration]. PsychOpen. https://onderzoekmetmensen.nl/nl/trial/52298


GülpenJ.
LegemaatA. M.
van DisE. A. M.
GeurtsenG. J.
WekkingE. M.
BurgerH.
DenysD. A. J. P.
BocktingC. L. H.
 (2026S). Supplementary materials to "No cognitive scarring over 20 years in recurrent depression: Bidirectionality of cognitive functioning and depression"
[Supplementary tables and figures]. PsychOpen. 10.23668/psycharchives.22291


## Data Availability

The corresponding author JG had full access to the study data and materials. The data, materials and codes used for this article may be shared on reasonable request to the corresponding author.

## Social Media Accounts

*Joost Gülpen:* LinkedIn

## Preregistration

Before start of the follow-up study, it was registered at the Overview of the Medical Research in the Netherlands (OMON), the former Dutch Trial Register, under ID (NL-OMON52298) (Gülpen et al., 2021S).

## Reporting Guidelines

Clinical trial, intervention study follows the CONSORT statement.
